# THE IMPORTANCE OF PERSONALITY TRAITS FOR COGNITIVE HEALTHSPAN

**DOI:** 10.1093/geroni/igad104.1304

**Published:** 2023-12-21

**Authors:** Tomiko Yoneda, Tristen Lozinski, Eileen Graham, Andrea Piccinin, Graciela Muniz-Terrera

**Affiliations:** University of California, Davis, Davis, California, United States; University of Regina, Regina, British Columbia, Canada; Northwestern University, Chicago, Illinois, United States; University of Victoria, Victoria, British Columbia, Canada; Ohio University, Athens, Ohio, United States

## Abstract

The existing literature consistently finds that personality traits are associated with dementia and with risk of mortality. However, few studies have simultaneously examined the impact of traits on dementia while accounting for death as a competing risk, which is critical as dementia and mortality are dependent outcomes and the likelihood of each increases alongside older age. In response, data were drawn from the Rush Memory and Aging Project (N=1954; baseline mean age=80 years; 74% female; up to 23 annual assessments). We used multi-state survival models to investigate whether traits (conscientiousness, extraversion, and neuroticism) were associated with transitions between cognitive status categories (No Cognitive Impairment, NCI; Mild Cognitive Impairment, MCI; dementia) and death. Further, multinomial regression models estimated cognitive healthspan (i.e., years without cognitive impairment) and total longevity. Adjusting for demographics, depressive symptoms, and APOEε4, personality traits were most important in the transition from NCI to MCI; higher conscientiousness was associated with a ~22% decreased risk of transitioning from NCI to MCI (HR=0.78, 95%CI=0.72, 0.85), whereas higher neuroticism was associated with a ~12% increased risk of transitioning from NCI to MCI (HR=1.12, 95%CI=1.04, 1.21). Life expectancy analyses suggested that participants who were higher in conscientiousness were estimated to live longer without cognitive impairment compared to individuals lower in conscientiousness. Findings were similar, though less pronounced, for individuals higher in extraversion or lower in neuroticism. Importantly, none of the traits appeared to be important for total longevity. Together, these results highlight the importance of personality traits, particularly conscientiousness, for maximizing cognitive healthspan.

